# Identification of the LH surge by measuring intact and total immunoreactivity in urine for prediction of ovulation time

**DOI:** 10.1007/s42000-022-00368-9

**Published:** 2022-05-26

**Authors:** And Demir, Matti Hero, Henrik Alfthan, Amro Passioni, Juha S. Tapanainen, Ulf-Håkan Stenman

**Affiliations:** 1 Pediatric Research Center, New Childrenʼs Hospital, University of Helsinki and Helsinki University Hospital, Biomedicum II, 6th floor, Tukholmankatu 8 A, FIN-00290 Helsinki, Finland; 2 HUSLAB, Helsinki University Hospital, Helsinki, Finland; 3 Department of Clinical Chemistry, Helsinki University Hospital, Helsinki, Finland; 4 Department of Obstetrics and Gynecology, University of Helsinki and Helsinki University Hospital, Helsinki, Finland; 5 Department of Obstetrics and Gynecology, Medical Research Center, PEDEGO Research Unit, University of Oulu and Oulu University Hospital, Oulu, Finland

**Keywords:** Urinary, Luteinizing hormone, Gonadotropin, Menstruation, Ovulation, Fertility, Infertility, Ovulation predictor kit

## Abstract

**Objectives:**

In our earlier study, we separated three different molecular forms of urinary LH-ir (U-LH-ir) by gel filtration and identified them by immunoassay in urine from regularly menstruating women on periovulatory days. U-LH-ir is composed of intact luteinizing hormone (LH), its free beta-subunit (LHβ), and the core fragment of LHβ (LHβcf), the latter two establishing the non-intact portion of LH-ir. The aim was to determine whether timing of ovulation can be improved by detecting different molecular forms of U-LH-ir in women of reproductive age.

**Methods:**

We determined intact and total U-LH-ir in 14 regularly menstruating women on consecutive periovulatory days during the menstrual cycle. Non-intact LH-ir was calculated as the arithmetic difference between total and intact LH-ir. In addition, LH-ir was determined in both serum and urine from four of the women throughout the menstrual cycle.

**Results:**

During the LH surge, U-LH-ir consisted mainly of intact LH and presented with an abrupt increase. Intact U-LH-ir dropped rapidly within 1 day after the surge, reaching baseline levels at the end of the luteal phase. In contrast, LHβcf in urine increased further 1 day after the surge. After this, most of the U-LH-ir consisted of LHβcf and it remained strongly elevated (over fivefold compared to intact LH) for the first 3 days after the LH surge, moderately elevated (over threefold) thereafter until day + 5, and mildly elevated until day + 7.

**Conclusions:**

Total and non-intact LH-ir are potential add-on characteristics which can be utilized in ovulation predictor kits to measure LH-ir in urine beyond the LH surge during a broader time frame, thereby paving the way for more precise prediction of the timing of ovulation than that obtained with currently available products.

## Introduction

Luteinizing hormone (LH) and human chorionic gonadotropin (hCG) are highly homologous and have the same bioactivity. They are mainly eliminated through the kidneys. During renal excretion, the two subunits, α and β, are separated and are partially degraded to so-called core fragments. LH and hCG are highly homologous and their subunits are further degraded by proteolytic cleavage in similar ways. The LHβ core fragment (LHβcf) contains amino acids 6–40 and 49–93, while the hCGβ core fragment (hCGβcf) contains amino acids 6–40 and 6–40 and LHβ 55–92 [1]. When isolated from urine, LHβcf is microheterogeneous, with molecular weights of 10,000–11,000 kilodaltons. The structure of LHβcf is not, in fact, precisely known, but, based on its reactivity with different LHβ and hCGβ antibodies, it resembles hCGβcf. Birken et al. isolated 12 kDA-sized LHβ core fragments from the pituitary and showed that they are formed by proteolytic digestion of LHβ, which causes losses in N- and C-terminal parts of LHβ [1]. Birken and Kovalevskaja have developed monoclonal antibodies and specific sandwich immunoassays based on these. LH shares epitopes with LHβ, LHβcf, hCG, hCGβ, and hCGβcf [2; 3]; thus, development of immunoassays is demanding. Furthermore, a common variant of LH is not recognized by some monoclonal antibodies.

A rapid increase in LH plasma, called an LH surge, induces ovulation within 2–3 days. The surge can be detected by the observation of LH in serum or in urine with ovulation predictor kits (OPKs). If a woman knows the duration of her menstrual cycle, she can plan the timing of testing. The average length of the menstrual cycle is 28 days, but a regular cycle lasting anywhere between 24 and 38 days is considered normal [4; 5; 6]. The cycle length is determined by follicular growth and by the lifespan of the corpus luteum [7]. Many women experience varying cycle lengths with back and forth shifts in the day of ovulation, which may pose a problem especially for infertility patients. To detect ovulation, patients are at present required to determine LH levels in urine with an OPK daily until getting a positive result, which causes undue stress in addition to the financial burden [8; 9].

OPKs may miss the LH surge for various reasons, one being an unusually short cycle. These problems compromise the reliability of ovulation timing by couples being treated for infertility. Only a fraction of apparently ovulatory cycles result in the development of a viable conceptus after appropriately timed intercourse. Wilcox et al. concluded that daily acts of intercourse during the potentially fertile period failed to produce a viable pregnancy in approximately 66% of presumed ovulatory cycles based on hormonal indices [10]. The window of opportunity for fertility is affected by uncertainty about the time of ovulation, the lifespan of the ovum and sperm in the female reproductive tract, and the contribution of other factors [11].

Better timing of intercourse can improve the probability of achieving pregnancy, and a majority of commercially available OPKs reliably detect the LH surge, which heralds imminent ovulation [12]. Because a positive urine test for the LH surge precedes ovulation by 1–2 days, it is possible to use OPKs for planning timed intercourse and intrauterine insemination. Randomized controlled trials show that the use of OPKs may increase pregnancy rates [13]. Thus, OPKs are regarded as the best method to predict ovulation [14]. Use of OPKs that also detect estrone-3-glucuronide (E3G) facilitates prediction of the onset of the fertile period before the LH surge [15; 16; 17].

The fertility window begins approximately 3–5 days (sperm lifespan) before ovulation and continues for approximately 1–2 days (oocyte lifespan) after ovulation [18]. Onset of the LH surge precedes ovulation by 35–44 h, and the peak level of serum LH (S-LH) precedes ovulation by 10–12 h [19; 20]. Pregnancy rates are highest from 2 days before ovulation to the day of ovulation [9].

We have recently described methods for separation and determination of three molecular forms of LH immunoreactivity (LH-ir), namely, intact LH, its free beta-subunit (LHβ), and LHβcf. The latter two together form the non-intact portion of the LH-ir. The three forms can be separated by gel filtration of urine obtained from regularly menstruating women, LHβcf being the dominant form among degradation products of LH subsequent to the LH surge [21].

In the current study, we have examined whether specific detection of the various forms of LH in urine can improve detection of the LH surge. We measured the concentrations of the various forms of LH in daily urine and serum samples throughout the menstrual cycle by employing assays that separately detect intact and total LH immunoreactivity (LH-ir). Currently, it is not possible to determine LH concentrations specifically for LH-beta (LHβ) or its core fragment (LHβcf) by any commercially available assay. Thus, we employed two different immunofluorometric LH assays, one that measured only intact LH and the other capable of detecting “total LH,” i.e., intact LH, LHβ, and LHβcf. LH-ir originating from wild-type and variant LH molecules was detectable by both assays [22; 23; 24].

## Materials and methods

### Samples and storage conditions

The study was performed at the Department of Clinical Chemistry, Helsinki University Hospital, Helsinki, Finland. Urine samples were obtained from 14 healthy, 22–48-year-old, regularly menstruating women on consecutive periovulatory days, i.e., about 1 week before and after the estimated day of ovulation according to previous cycles. The LH surge was defined as a twofold or higher increase in urine concentrations of intact LH on consecutive days. Total and intact LH-ir were measured separately by the respective assays in both serum and urine throughout the menstrual cycle in four of the women. Serum samples were obtained for about 1 week before and after the estimated day of ovulation according to previous cycles in order to confirm the day of LH surge based on urinary intact LH, as outlined above. For each participant, days were numbered according to the number of days before ( −) and after ( +) the LH surge. The women did not receive any medication 3 months prior to and during the study, including hormonal contraceptives and hormonal intrauterine devices. Serum samples were stored at − 20 °C, and, based on our earlier findings, urine samples were stored at 4 °C for less than 1 week before assayed.

### Immunofluorometric assays

The immunofluorometric methods (IFMAs) are commercial sandwich assays using monoclonal antibodies (LH Delfia® and LHspec Delfia®, PerkinElmer Wallac, Turku, Finland). One antibody is immobilized onto a microtiter strip well and the other is labeled with a europium chelate. In the assay for intact LH, the capture antibody is specific for LHβ and the detection antibody for the α-subunit. In the LHspec assay, both capture and detection antibodies are directed to β-subunit targeting specifically different epitopes [22]. The assay for intact LH also measures hCG [25]. Because urine of menstruating women contains negligible amounts of hCG [26], the LH immunoreactivity measured by this assay is intact LH. In contrast, the LHspec assay has been designed to detect total LH, i.e., intact LH, LHβ, and LHβcf. The assays were performed according to the instructions of the manufacturer. A sample volume of 25 µL was used for serum and urine. The total assay volume was 225 µL. The calibrators were standardized against the WHO 2nd International Standard for pituitary LH for immunoassay (code 80/552) [27]. Total LH concentration is the sum of the concentrations of U-LH, U-LHβ, and U-LHβcf; therefore, non-intact U-LH concentration was calculated as the arithmetic difference in concentration between total and intact U-LH.

### Buffers

TBS buffer contained 0.05 mol/L Tris–HCL, pH 7.7, 0.15 mol/L NaCl, and 0.5 g/L NaN3. The assay buffer in the IFMAs was TBS containing 5 g/L bovine serum albumin (BSA), 0.5 g/L bovine globulin, 0.05 g/L Tween 20 (Sigma-Aldrich), and 20 mg/L DTPA. The wash solution was obtained from PerkinElmer Wallac, Turku, Finland.

### Statistics

The paired-samples *t*-test was used to analyze differences in the ratios of non-intact to intact LH immunoreactivity between adjacent days of the menstrual cycle.

## Results

**Fig. 1:**
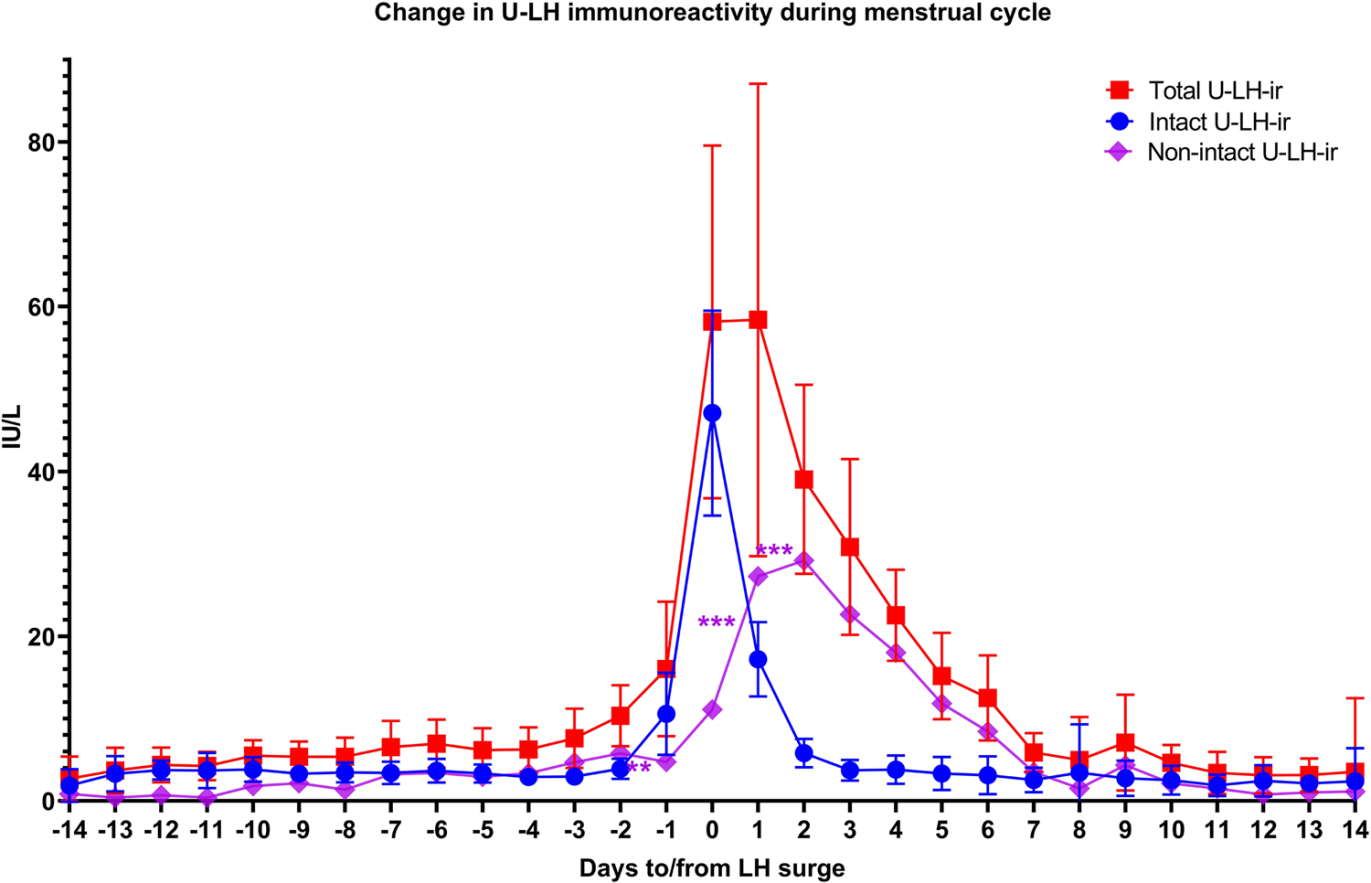
Concentrations of intact, non-intact, and total LH immunoreactivity (LH-ir) as well as 95% confidence intervals for total and intact LH determined in urine from 14 subjects during different days of the menstrual cycle. Statistically significant changes in non-intact LH-ir are denoted as follows: **P < 0.01, ***P < 0.001

**Fig. 2:**
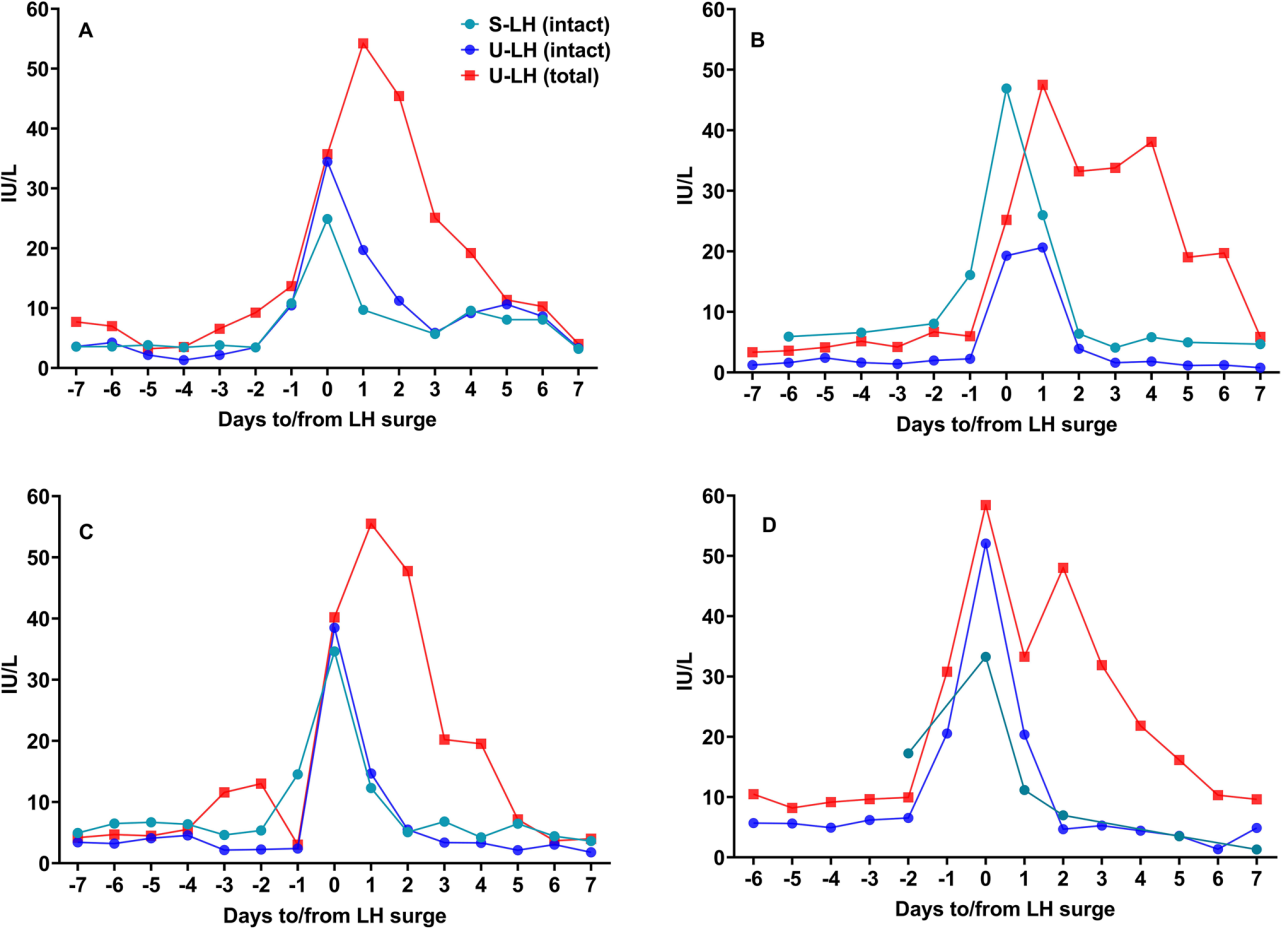
Concentrations of intact, non-intact, and total LH in serum and urine samples during the course of an additional full menstrual cycle in four subjects

**Fig. 3:**
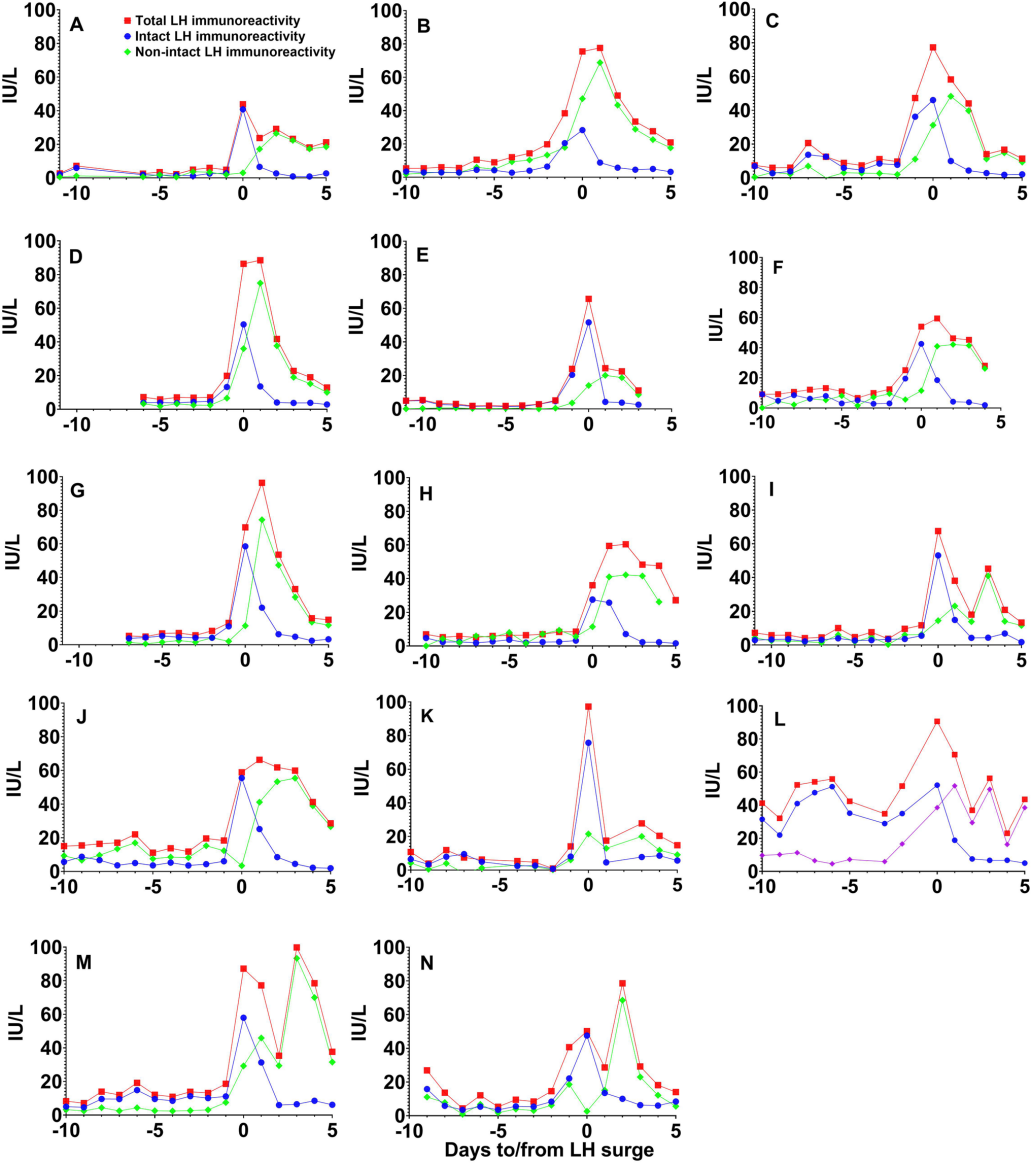
Total LH, intact LH, and non-intact LH (LHβ or LHβcf) immunoreactivity measured in daily urine samples taken during different days of the cycle in 14 women of reproductive age. Symbols and legends depicted in panel **A** are valid also for all the other panels

**Fig. 4:**
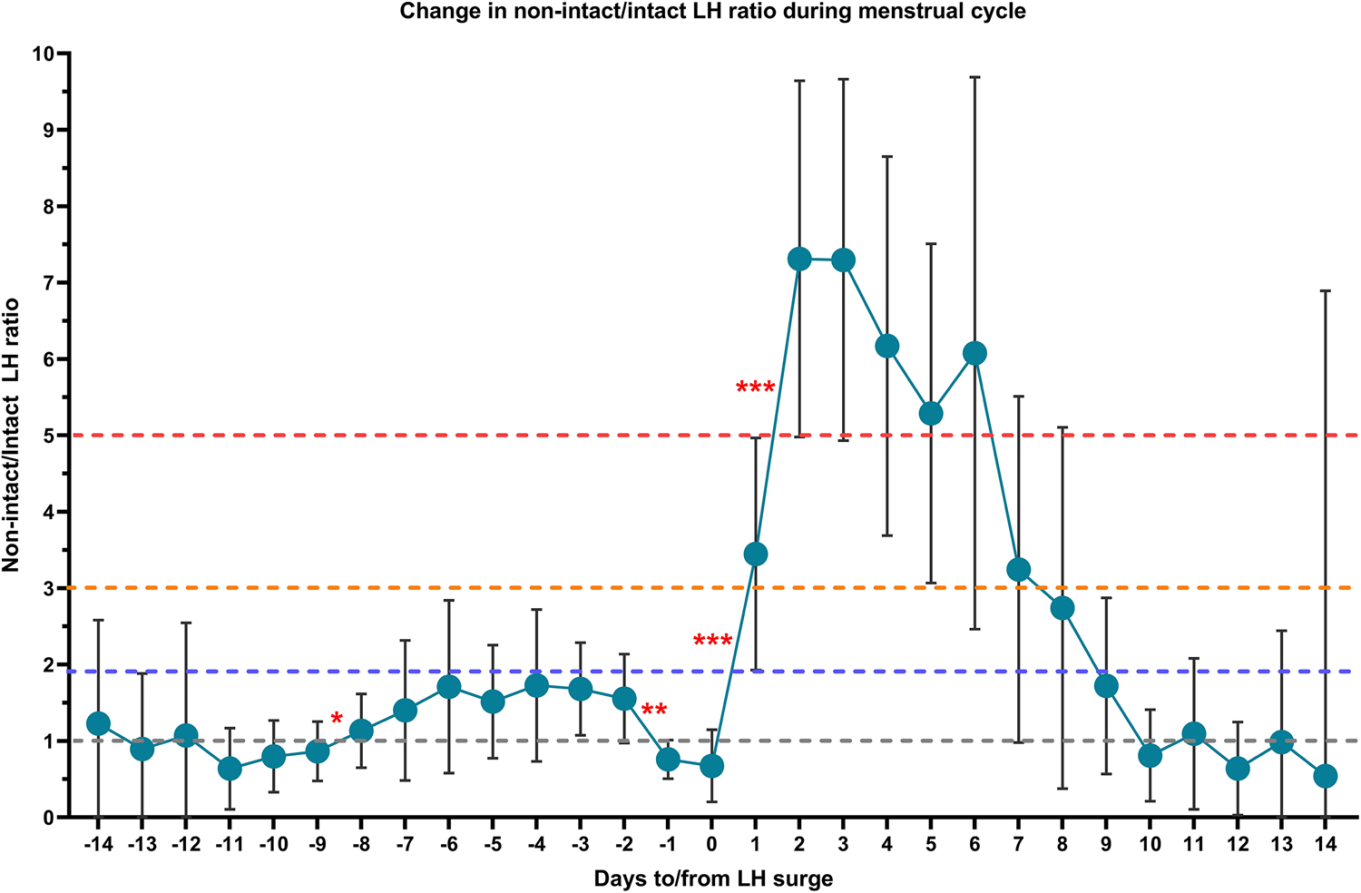
Course of the changes in urinary concentrations of non-intact LH in relation to intact LH during the menstrual cycle for all 14 subjects (circles depict the mean ratios of non-intact to intact U-LH-ir immunoreactivity, and bars represent the 95% confidence intervals). Statistically significant changes are denoted as follows: *P < 0.05, **P < 0.01, ***P < 0.001

Figure 1 shows intact and total U-LH-ir during 2 weeks before and after the LH surge. During the follicular phase (days − 2 to − 7), the concentrations of U-LH were low (Fig. 1); total U-LH concentrations ranged between 4 and 8 IU/L, and were about twofold as high as those of intact U-LH (Figs. 2 and 3). The concentrations of intact and total U-LH started to increase on day − 1 and increased at the same rate as those of S-LH. The concentrations of S-LH and intact U-LH were very similar, confirming that LH-ir in serum (S-LH-ir) is derived mainly from intact LH. Levels of intact U-LH-ir and S-LH-ir exhibited a relatively parallel course together during the rise as well as during the steep decrease within the next 2 days following the LH surge. After the peak of intact U-LH-ir, the concentrations of total U-LH increased further on day + 1, after which the concentrations decreased slowly as of day + 2 until reaching baseline within 1 week (Fig. 1). After the surge, the concentrations of total U-LH were much higher than those of intact U-LH (Figs. 2 and 3). On the day of the LH surge, the concentrations of intact and total U-LH were similar (Fig. 1). After the LH peak, non-intact U-LH-ir kept increasing during days + 1 and + 2 (Figs. 1 and 4) due to continuing excretion of degradation products of LH, hence, of LHβ and LHβcf, which constituted the main forms of LH-ir also during post-surge days + 3 to + 6 (Figs. 1, 2, 3, 4) [21].

There was no statistically significant difference between serum concentrations of total and intact LH in four of the women who provided daily blood samples in addition to urine samples (*P* < 0.001). Thus, neither LHβ nor LHβcf was detectable in serum at any stage of the cycle. The patterns of intact and total U-LH-ir around the LH surge in individual menstrual cycles of the 14 women are shown in Fig. 3. In all women, the peak of intact LH was excreted as a single peak, while total LH occurred as two peaks in six cases and as a broad peak in seven cases.

The peak in total U-LH concentrations that was observed on day + 1 (Fig. 1) was due to the rapid increase in non-intact U-LH-ir (Fig. 4). The ratio of non-intact to intact U-LH-ir during the course of periovulatory days in all 14 subjects is depicted in Fig. 4. This ratio showed a significant drop between days − 2 and − 1 before the day of the LH surge (*P* < 0.01), remaining at or below 1 on the day of LH surge. The latter ratio rose significantly immediately after the LH peak, the mean value increasing from below 1 to over 3 within 24 h (*P* < 0.001). The difference was more than twofold on day + 1 (*P* < 0.001) and fivefold on day + 2 (*P* < 0.001); the mean value reached 3.4 on day + 1 and 7.3 on days + 2 through + 3 (*P* < 0.001 for both). The mean ratio of non-intact to intact U-LH-ir remained above 5 on days + 4 through + 6, as this ratio persisted at levels over 3 on days + 4 through + 5 and over 2 on day + 6 in all cases. After the LH surge, the dominance of non-intact over intact U-LH-ir lasted at least until day + 7 (Fig. 4).

## Discussion

The results of our study provide new information of the metabolism of LH in women of reproductive age. Timing of the LH surge in blood is very similar when based on intact LH and LHβ in urine, but the concentrations of LHβcf show a different pattern: after the surge, U-LH-ir consists of mainly non-intact LH (Figs. 1 and 3), and for the most part LHβcf [21; 28]. Intact U-LH-ir drops to baseline levels within 1–2 days after the surge, while the level of non-intact U-LH-ir keeps increasing 1–2 days after the LH surge and returns to baseline before day + 7, as observed also in total U-LH-ir levels (Figs. 1, 2, 3). This indicates that the degradation of LH is a slow process that takes place in the kidneys and possibly in other organs.

The metabolism of the corresponding fragment of hCG, i.e., hCGβcf, shows a similar pattern [29]. This is most likely a result of uptake of hCG by the tubules of the kidney, where it is partially degraded by proteolytic enzymes [30; 31; 32]. The core fragment of hCG is resistant to further degradation because it contains a tight knot held together by six disulfide bonds and is excreted after a delay of 1 or several days [30; 31; 32]. LHβcf is also excreted over several days after the LH surge [21; 28]. Interestingly, LHβ is also excreted during the surge and disappears at the same time as LH; however, it is not detected in plasma, as noted above, and this indicates that it is formed by partial digestion of intact LH during the excretion of LH through the kidney tubules. In the light of earlier studies, degradation products of LH do not occur at detectable levels in serum [1; 2; 21]. Overall, these data prove that the fertility window obtained by measuring intact (or total) LH in serum is narrower than that observed by measuring non-intact (or total) LH in urine.

The concentrations of intact U-LH were higher than those of non-intact U-LH before and on the day of the LH surge; however, immediately after the LH surge, intact U-LH-ir levels decreased rapidly, while non-intact LH-ir remained high. As a result, the concentration of non-intact LH-ir in urine remained much higher than that of intact LH-ir (over fivefold) for the first 3 days after the LH surge, moderately elevated (over threefold) thereafter until day + 5, and mildly elevated until day + 7. This is in line with the findings of our earlier study in which non-intact LH-ir formed about 90% of LH immunoreactivity in urine 2–5 days after the LH surge [21].

The current study is the first to demonstrate the broader extent of urinary excretion of non-intact LH, hence of LHβcf, after the LH surge in 14 menstruating women by commercially available assays (Figs. 1, 2, 3), confirming the earlier preliminary findings reported by us and O’Connor et al. [21; 28]. Significant increases in non-intact to intact LH ratio above 3 on day + 1 and above 5 on day + 2 point to an intensified expression of LHβcf immunoreactivity in urine during the highest probable window of ovulation, which current OPKs fail to detect to this extent. If the “fertility window” is defined on the basis of total or non-intact LH-ir in urine, it is broader than that detected by intact LH. Further research is required to determine whether this would improve the chances for conception.

Earlier studies have shown that the S-LH peak may precede the U-LH peak and demonstrated an atypical, biphasic, or plateau-type LH peak during several days after the LH surge (as in Figs. 1 and 2) [33]. However, our results illustrate that if LH is measured by a method that detects only intact LH, the peak concentrations of LH in urine and serum occur at the same time (Fig. 1). If a method for total LH is used, the peak in urine may be biphasic or broad. A double peak of intact LH was not seen in serum, nor was it seen in non-intact LH. This suggests that non-intact LH is formed in the kidneys and excreted into urine for several days after the LH surge.

Knowledge regarding the period of high fertility prior to ovulation, which was previously believed to cover the 5 days prior to ovulation plus the day of ovulation [10; 11; 34; 35; 36], has been updated. Thus, it is now known that it starts with a preovulatory period of up to 4 to 7 days and continues with a postovulatory period of up to 2 days, which has been established through relatively more recent findings, as outlined in a publication by Wilcox et al. [37]. Indeed, an additional 1 day on average on top of the day of ovulation should be taken into consideration to allow for the lifespans of sperm and ovum (1.4 days and 0.7 days on average, respectively) in computing the range of the fertility window [18; 38; 39]. Based on these facts, it is justified to suggest incorporating separate detection of intact and non-intact U-LH-ir, the latter preferably in the form of LHβcf, in order to improve the performance of current OPKs. OPKs are designed as sandwich assays, but little is known about their specificity for various forms of LH.

Our results show that an OPK detecting total or non-intact U-LH-ir will be strongly positive for up to 3 days after the peak for intact U-LH-ir, which is positive for only a single day during the LH surge. Therefore, the likelihood of detecting the fertility window within and beyond the LH surge period should be greater if an OPK detecting total U-LH-ir rather than intact LH is used. The best alternative may be to use an OPK detecting both intact and non-intact U-LH-ir separately, the latter preferably in the form of LHβcf. An OPK that detects total and intact LH-ir can function as a compromise solution by calculating the non-intact LH-ir as the arithmetic difference. An OPK as outlined above would cover the whole fertility window and provide more detailed information.

However, the design of such an assay is challenging, as specific antibodies for LHβ and LHβcf are currently unavailable; thus, the specificities of OPKs for various forms of LH need to be examined. This would be feasible if standards for LHβcf were available, as is the case for various forms of hCG [2]. We may conclude that once these limitations have been adequately addressed, total or non-intact U-LH-ir are potential add-on characteristics that can be used in OPKs to detect changes in LH-ir during a broader time frame around the LH surge for a more precise prediction of the timing of ovulation than that achieved with currently available methods. Moreover, such OPKs should be tested further in clinical studies to resolve the issue of whether this would result in any advantage for the timing of intercourse or for in vitro fertilization.

## Abbreviations

FSH: Follicle-stimulating hormone
hCG: Human chorionic gonadotropin
hCGβcf: Core fragment of human chorionic gonadotropin
IFMA: Immunofluorometric assay
LH: Luteinizing hormone
LH-ir: LH immunoreactivity
LHβ: Beta subunit of luteinizing hormone
LHβcf: Core fragment of luteinizing hormone
OPK: Ovulation predictor kit
